# Decoding the Conformation of Polylactic Acid in Block Copolymer Micelles

**DOI:** 10.1021/jacs.5c19227

**Published:** 2026-01-28

**Authors:** J. Muñoz-López, G. M. Tuveri, V. Barbieri, M. Basile, V. Cosenza, C. D. Lorenz, L. Ruiz-Pérez, G. Battaglia

**Affiliations:** † Institute for Bioengineering of Catalonia (IBEC), Baldiri Reixac 10, 08028 Barcelona, Spain; ‡ Department of Organic and Inorganic Chemistry, Faculty of Chemistry, 16724 University of Barcelona, Martí i Franquès 1-11, 08028 Barcelona, Spain; § Department of Biomedicine, Faculty of Medicine, Universitat de Barcelona, Casanova 143, 08036 Barcelona, Spain; ∥ Department of Condensed Matter Physics, Faculty of Physics, University of Barcelona, Martí i Franquès 1-11, 08028 Barcelona, Spain; ⊥ Department of Engineering, King’s College London, London WC2R 2LS, U.K.; # Serra Húnter Fellow, Department of Applied Physics, Faculty of Physics, University of Barcelona, Martí i Franquès 1-11, 08028 Barcelona, Spain; ∇ Catalan Institution for Research and Advanced Studies (ICREA), Passeig Lluis Companys 23, 08010 Barcelona, Spain

## Abstract

Understanding how molecular features dictate the self-assembly of amphiphilic block copolymers into well-defined nanostructures is essential for the rational design of advanced soft materials. However, the large number of interdependent parameters involved, such as particle size, aggregation number, interfacial curvature, and molecular weight, makes it challenging to establish general design principles. Here we establish a scaling-based framework for PEG-*b*-PLA micelles with a fixed hydrophilic–hydrophobic ratio. Systematic variation of molecular weights enables precise control of micelle size and aggregation number, quantified by DLS, cryo-TEM, and MALS.

## Graphical abstract

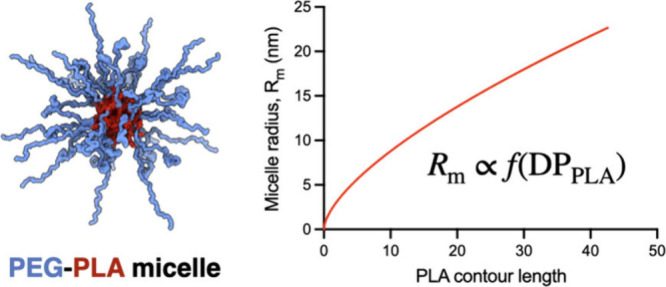

Polyethylene oxide-*block*-polylactide (PEG-*b*-PLA) diblock copolymers are among the most widely used building blocks in soft matter systems, exhibiting a broad range of self-assembled morphologies.^1^ Their widespread application in nanomedicine and biomedical fields is largely due to the high biocompatibility and nontoxicity of both blocks, as well as the degradability of the PLA segment.^2^ These features have contributed to the FDA approval of PEG-*b*-PLA copolymers for a wide range of medical applications.^3^ Consequently, PEG-*b*-PLA micelles have been widely explored as drug delivery systems, with numerous approaches leveraging smart nanoparticle design, particularly in the context of cancer therapy.^3–7^

Despite the widespread use of PEG-*b*-PLA micelle formulations, a persistent challenge in designing polymeric materials is the lack of a generalizable framework for predicting how molecular features translate into nanoscale structures.^8^ In this study, we address the complexity of these interdependent parameters by systematically investigating a series of PEG-*b*-PLA copolymers with controlled variations in both PEG and PLA block lengths, while maintaining a constant hydrophilic-to-hydrophobic ratio throughout the micelle series. This design strategy preserves the packing parameter and isolates the impact of both contour length and chain flexibility on micelle structure.

A series of well-defined PEG-*b*-PLA diblock copolymers was synthesized via ring-opening polymerization (ROP) of (±)-3,6-dimethyl-1,4-dioxan-2,5-dione, initiated by commercially available methoxy-PEG (mPEG-OH) of different molecular weights (2024, 5016, 10032, and 20064 Da) (Figure 1A). Each diblock copolymer maintained a hydrophilic-to-hydrophobic molar ratio of about 3:1, enabling systematic investigations of the effects of block chain length on micellization behavior and composition in aqueous solution.

**Figure 1:**
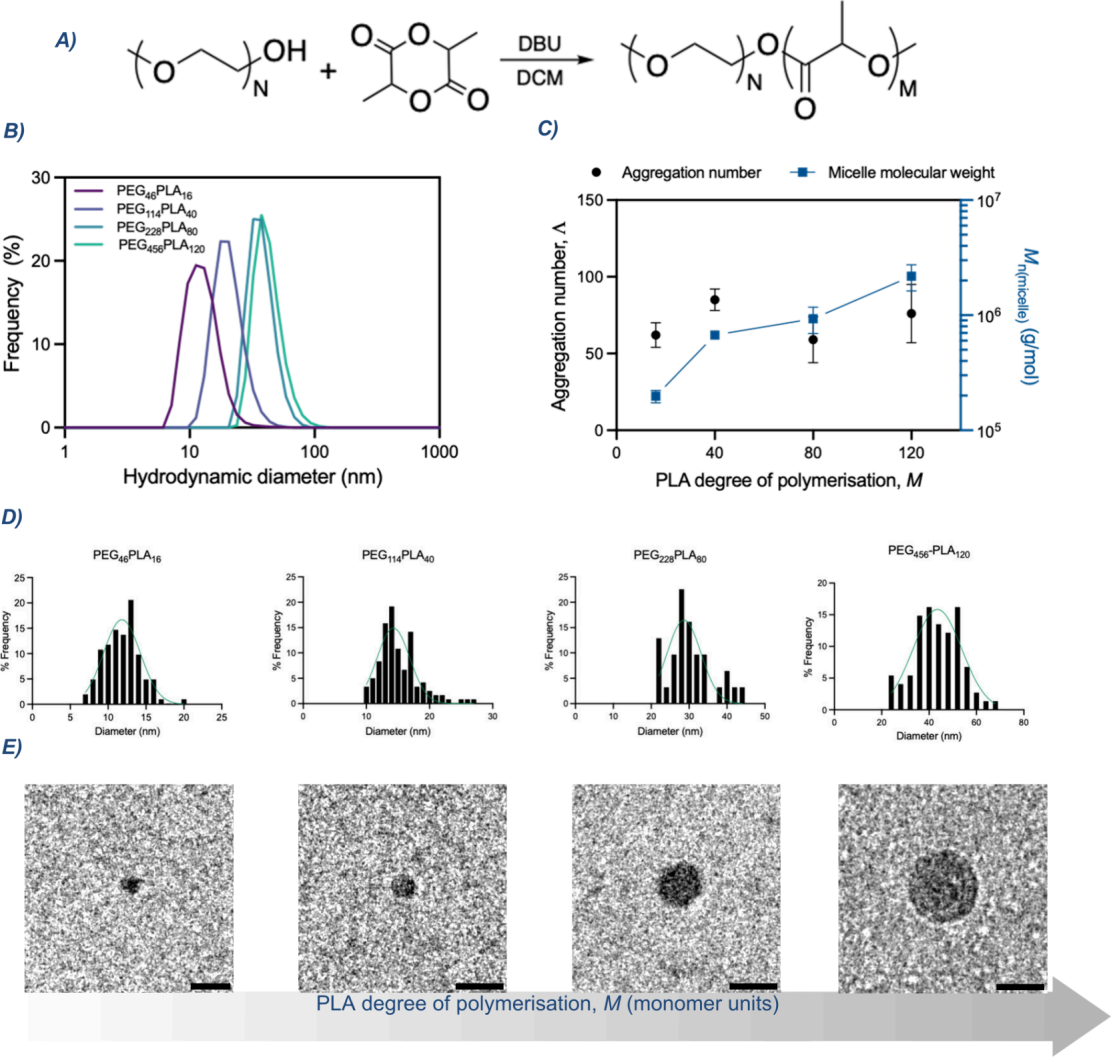
Characterization of PEG-*b*-PLA micelle library. (A) Schematic representation of PEG-*b*-PLA synthesis. (B) Number-based hydrodynamic diameter distribution of the micelles library as determined by DLS. (C) Quantification of micelle molecular weight and aggregation number, as measured by MALS. (D) Histograms of micelle size populations obtained from cryoTEM analysis and (E) cryoTEM micrographs of unstained PEG_*n*_-*b*-PLA_*m*_ micelles with increasing PLA degree of polymerization and LA monomer units. Scale bars are 30 nm.

The four sets of PEG-*b*-PLA diblock copolymers were characterized by both ^1^H NMR and GPC (Figures S3–S8). The GPC traces show a systematic shift toward shorter retention times with increasing molecular weight, consistent with successful chain extension from the PEG–OH initiators. In the case of PEG_114_-*b*-PLA_40_, two partially overlapping populations were observed, which originated from the bimodal molecular weight distribution of the commercial PEG initiator itself. Differential weight distribution plots and the steep slopes of the cumulative weight curves (Figure S8) indicated narrow molecular weight distribution for all samples, yielding dispersity indices *Đ* of 1.00, 1.03, 1.07, 1.00 for PEG_46_-*b*-PLA_16_, PEG_114_-*b*-PLA_40_, PEG_228_-*b*-PLA_80_, and PEG_456_-*b*-PLA_120_, respectively. Micelles were formed by the solvent-switch method involving the controlled and gradual addition of a DMF solution of the diblock copolymer to water under continuous stirring as previously reported,^9^ followed by overnight equilibration to ensure thermodynamic stabilization. The generated structures were characterized by cryogenic transmission electron microscopy (cryo-TEM) and dynamic light scattering DLS. Cryo-TEM confirmed the successful formation of spherical micelle morphologies, while DLS revealed a nonlinear increase in the hydrodynamic diameter *D*_h_ with increasing chain length (Figure 1B,E). The measured *D*_h_ values were 12.98 ± 4.42, 20.86 ± 4.66, 37.88 ± 9.44, 43.16 ± 4.88 nm for PEG_46_-*b*-PLA_16_, PEG_114_-*b*-PLA_40_, PEG_228_-*b*-PLA_80_, and PEG_456_-*b*-PLA_120_, respectively.

Frequency histograms derived from cryo-TEM measurements (Figures 1D and S9) enabled to calculate micelle sizes: 11.87 ± 2.35, 15.18 ± 3.22, 30.26 ± 5.76, and 43.1 ± 9.68 for the same series. The values obtained from both techniques are in strong correlation, with DLS consistently yielding larger diameters, as expected from the contribution of the hydrated PEG corona. Notably, the micelle contrast and contour definition in cryoTEM micrographs (Figure 1E) became progressively sharper with increasing PLA block length, consistent with enhanced segregation between the hydrophobic core and the hydrophilic corona. Although cryo-TEM revealed a more strictly monotonic increase in micelle size (Figure S10), the systematically smaller diameters measured by cryo-TEM are attributed to the limited electron contrast of the hydrated PEG corona, not contributing appreciably to the apparent micelle dimensions.

Multiangle dynamic light scattering MALS was employed to determine the molecular weight and aggregation number of the assemblies. As expected, the micellar molecular weight increased with the PLA degree of polymerization. However, the aggregation number (Λ) remained nearly constant across the series. The consistency of the MALS-derived molecular weights across independent replicates (Figure S11B) indicates that the micelles are not trapped in kinetically frozen states. Furthermore, since identical solvent conditions were used for all formulations, the minimal variation observed in micelles aggregation numbers can be attributed to the differences in polymer chain length rather than to solvent-driven effects.^10^ In contrast, these results suggest that the micelle core grows with PLA chain length without altering the number of constituent chains.

The nonlinear dependence between micelle radius and block chain length can be explained by different factors, such as (i) the coiling behavior of the hydrophobic block in the core, well described by polymer scaling laws;^11^ (ii) the combined steric and entropic penalties associated with crowding of hydrophilic chains in the corona, modulated by the polymer grafting density (σ); and (iii) the interfacial tension between hydrophilic and hydrophobic blocks, determined by the curvature of the generated structure. Thus, as shown in Scheme 1, the radius of a polymeric micelle, *R*_m_, can be broken down into the sum of two elements, namely, the core of the micelle (*R*_c_) and the brush height constituted by the micelle corona (*h*_p_):

1 $$R_{m}=h_{p}+R_{c}$$

**Scheme 1:**
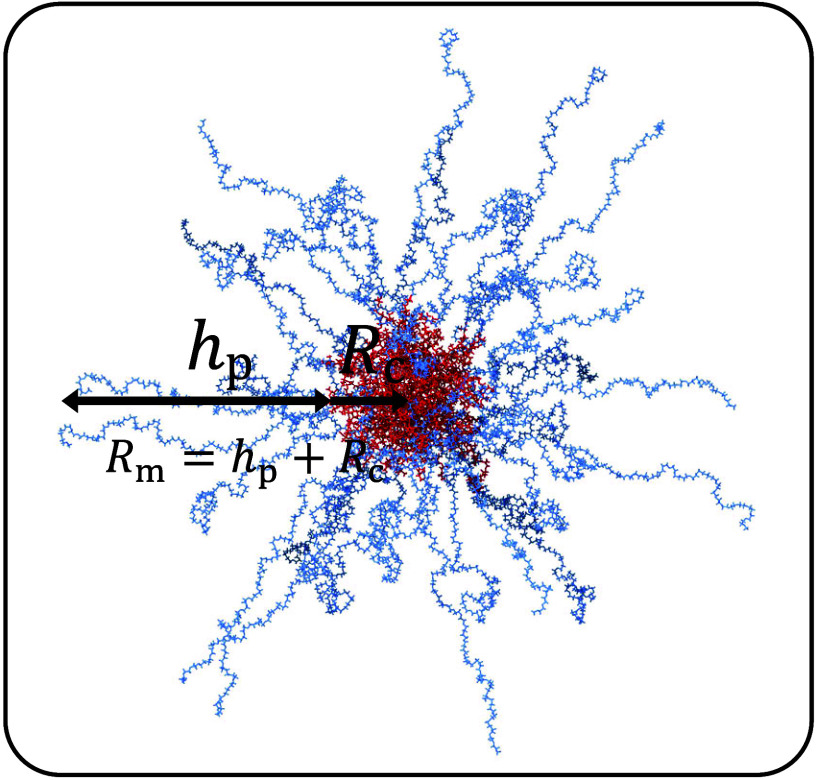
Schematic Model of a PEG_46_-*b*-PLA_16_ Micelle

The length of the hydrophilic blocks in curved substrates has been defined:^12,13^

2 $$h_{p}=R_{c}\left[{\left(1+\frac{\left(\gamma +2\right)N}{3R_{c}}{\left(\frac{va^{2}}{3\alpha_{0}}\right)}^{1/3}\right)}^{3/\gamma +2}-1\right]$$

where *N* is the degree of polymerization of the hydrophilic block, γ is a geometrical parameter that represents the packing of the chains at the surface, *a* is the size of a PEG monomer, *v* is the excluded volume of a PEG monomer, and α_0_ is the area per chain at the interface. It is important to note that the length of the hydrophilic block in the corona (*h*_p_) is not independent of the micelle core radius (*R*_c_), as chain stretching in the corona is influenced by the curvature, chain grafting density, and size of the hydrophobic core.^12^

The micelle core is inherently shaped by the coiled conformation adopted by the PLA hydrophobic block and the packing of the polymer chains in the confined space. The core radius *R*_c_ can be approximated as the end-to-end distance of the PLA block, extending from its first monomer, positioned at the center of the micelle, to its terminal monomer, at the interface with the adjacent PEG segment. Thus, theoretical estimations on the core radius can be based on end-to-end chain distance models that account for the conformational freedom and chain stiffness of the PLA polymer chain. To this end, we employed the worm-like chain model (WLC),^14^ which accounts for semiflexible polymer behavior by introducing the persistence length as a measure of the polymer’s intrinsic stiffness.

According to the WLC model, the mean-squared end-to-end distance is defined by the following equation:^11^

3 $$\left\langle r^{2}\right\rangle =2L_{p}L\left[1-\frac{L_{p}}{L}\left(1-e^{-L/L_{p}}\right)\right]$$

where *L*_p_ is the PLA polymer persistence length and *L* is the PLA contour length, defined as

4 L=bM

where *b* is the PLA monomer length and *M* is the degree of polymerization of the PLA block.

The persistence length *L*_p_ of the PLA polymer block within the micelle core was estimated by fitting the WLC model to the experimental hydrodynamic radii of the PEG-*b*-PLA micelle library under the three distinct regimes (see the Supporting Information (SI)). The fit for the rigid-rod model consistently overestimates the micelle radius, particularly for micelles formed with short PLA blocks. In contrast, both semiflexible and flexible WLC models yield physically meaningful and quantitatively accurate fits across the full micelle series. The semiflexible fit produced a persistence length *L*_p_ = 0.98 nm (95% CI: 0.66–1.39 nm) with a coefficient of determination *r* ^2^ = 0.97 and a symmetrical standard error of 0.1. The flexible chain limit (Figure 2B) also showed an excellent correlation with the data (*r* ^2^ = 0.98), returning a persistence length of *L*_p_ = 0.82 nm (95% CI: 0.47–1.29 nm) and a standard error of 0.02. Both regimes are well-aligned with the observed stretched-to-coil transition behavior from the SAW model (see SI section 2.1).

**Figure 2:**
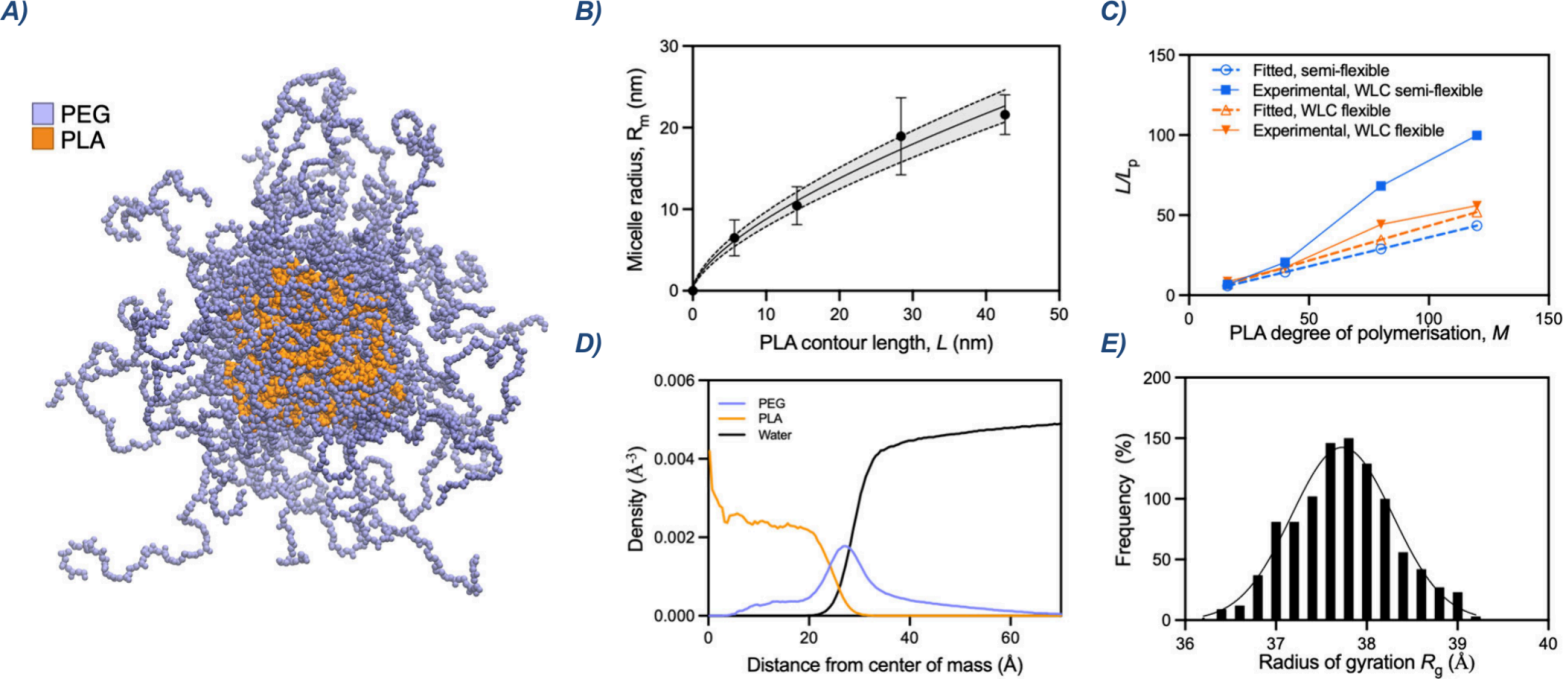
(A) Coarse-grained model of a PEG_46_-*b*-PLA_16_ micelle shown in its relaxed equilibrium configuration. (B) Nonlinear fit of WLC model in the flexible regime. (C) Comparison between fitted and experimentally determined persistence lengths for both semiflexible and flexible regimes. (D) Radial density profiles of the coarse-grained PEG_46_-*b*-PLA_16_ micelle showing the spatial distribution of the hydrophilic (PEG) and hydrophobic (PLA) blocks relative to the micelle center. (E) Histogram of the radius of gyration distribution for the coarse-grained PEG_46_-*b*-PLA_16_ micelle.

To further validate the theoretical *L*_p_ estimates from nonlinear regression, we independently estimated experimental *L*_p_ values for each micelle formulation using the aggregation number-based expression derived for the WLC model. For the semiflexible regime, the relationship is given by

$$\Lambda \left(L\right)=J\frac{b}{L}{\left[2L_{p}L-2L_{p}^{2}\left(1-e^{-L/L_{p}}\right)\right]}^{3/2}$$ 5

and that for the flexible regime is

6 $$\Lambda \left(L\right)=Jb{\left(2L_{p}\right)}^{3/2}L^{1/2}$$

where *J* = (4πρ*N*_A_/3*m*_0_) ≈ 42.05 nm^–3^.

The experimentally determined persistence lengths for each micelle formulation under the flexible regime fell within the 95% confidence interval of the fitted *L*_p_ value obtained from nonlinear regression. This finding helped to reinforce the consistency and robustness of the model in the flexible limit. Conversely, larger discrepancies are observed in the semiflexible regime, particularly as the PLA contour length increases (Figure 2C).

Taken together, the data discussed above indicate that the increasing conformational flexibility of the PLA block with chain length, combined with the entropically relaxed behavior of the PEG corona, governs the observed micellar size scaling (see SI section 2.1). We therefore conducted a coarse-grained molecular dynamics simulation of a micelle composed of the shorter polymer series, PEG_46_-*b*-PLA_16_ (Figure 2A) to validate this hypothesis. Despite its short PLA chain contour length, this formulation exhibited the highest stiffness in the experimental data set, making it a suitable reference point for examining structural organization under semiflexible and flexible conditions. From the simulation trajectory, we extracted the radial density profiles of the PLA and PEG blocks, as well as the solvation water, relative to the micelle’s center of mass (Figure 2D). As expected, the water density profile sharply increases between 20 and 30 Å, clearly delineating the boundary between the hydrophobic and the hydrophilic corona.

The PLA density reaches its maximum near the center of the micelle and displays a broad, flat plateau between 5 and 20 Å, indicating that PLA monomers are uniformly distributed across the core volume. This uniformity is characteristic of coiled, entangled conformations rather than radially stretched chains.^15^ Combined with the experimentally determined Flory exponent (Figure S1), this suggests that PLA chains are neither fully extended nor ideally coiled for a polymer melt, but instead occupy an intermediate regime of partial extension, consistent with wormlike chain behavior. The gradual decay of the PLA density between 20 and 30 Å suggests that some chains extend toward the core–corona interface, increasing the effective interfacial surface area beyond that of an idealized spherical core. This trend, when viewed alongside the increase in micelle radius and chain flexibility observed for longer PLA blocks, suggests a more diffuse PLA interfacial profile in larger micelles.

The radial distribution of the PEG block shows a broad, asymmetric peak centered around 30 Å, with a smooth rise from the core–corona interface and a gradual decay into the bulk solvent, which is consistent with the trend observed in PEG–PLGA micelles.^16^ The presence of nonzero PEG density near the micelle core indicates that PEG segments remain close to their PLA junction points, consistent with coiled chain conformations. This behavior is facilitated by the increased interfacial area per chain, which reduces steric repulsion and allows PEG chains to relax more into disordered, entropically favorable configurations, resulting in the diffuse, partially hydrated corona as observed by cryo-TEM.

Regarding micelle size, the hydrodynamic radius *R*_h_ obtained from simulation is 6.9 nm, showing excellent agreement with the experimental value measured by dynamic light scattering and cryo-TEM. Due to the small size of the micelles, experimental determination of the radius of gyration *R*_g_ is particularly challenging. In the literature, isotropic scattering becomes unreliable for particles below a certain size threshold, making precise *R*_g_ measurements difficult in this regime.^17^ Nevertheless, the theoretical *R*_g_ distribution obtained from simulations (Figure 2E) reveals a narrow peak centered around 37.9–38.1 Å, indicating a stable micellar structure throughout the trajectory.

In conclusion, our findings contribute to a predictive framework for block copolymer micelle design by bridging molecular-scale inputs with nanoscale structure. The identification of a unifying molecular descriptor reduces the nanoparticle engineering to the design of the unimer itself. This offers a transferable strategy for engineering polymer self-assembly in soft materials, drug delivery, and nanomedicine.

## Supplementary Material



## Notes

Additional experimental details, materials and methods, polymer synthesis and characterization data, mathematical derivation of the theoretical equations, and supporting analyses underlying the conclusions of this work (PDF)
