# Supplementary material for: Decoding the Conformation of Polylactic Acid in Block Copolymer Micelles
Source: J Am Chem Soc. 2026 Jan 28;148(5):4839–43. doi: 10.1021/jacs.5c19227 (PMC12903854; doi:10.1021/jacs.5c19227)
Supplement: Supplementary file 1 [file ja5c19227_si_001.pdf]

Supporting information

Decoding the conformation of polylactic acid in block copolymer micelles

J. Muñoz-López[1,2], G. M. Tuveri[1,4], V. Barbieri[1], M. Basile[1,3], V. Cosenza[1], C. D. Lorenz[5], L. Ruiz-Pérez[1,6]*, G. Battaglia[1,7]*.

[1] Institute for Bioengineering of Catalonia (IBEC), Baldiri Reixac 10, 08028, Barcelona

[2] Department of Organic and Inorganic Chemistry, Faculty of Chemistry, University of Barcelona, Martí i Franquès 1-11, 08028, Barcelona

[3] Department of Biomedicine, Faculty of Medicine, Universitat de Barcelona, Casanova 143, 08036, Barcelona

[4] Department of Condensed Matter Physics, Faculty of Physics, University of Barcelona, Martí i Franquès 1-11, 08028, Barcelona

[5] Department of Engineering, King's College London, London, WC2R 2LS, UK.

[6] Serra Húnter Fellow, Department of Applied Physics, Faculty of Physics, University of Barcelona, Martí i Franquès 1-11, 08028, Barcelona

[7] Catalan Institution for Research and Advanced Studies (ICREA), Passeig Lluis Companys 23, 08010, Barcelona

Materials and General methods

α-methoxy-ω-hydroxy-poly(ethtylene glycol) (mPEG-OH) with number-average molecular weights (M_n) of 2024, 5016, 10032, and 20064 g/mol; 1,8-diazabicyclo[5.4.0]undec-7-ene (DBU); anhydrous tetrahydrofuran (THF); anhydrous dichloromethane (DCM); methanol (MeOH); sodium azide, 2,6-Di-tert-butyl-4-methylphenol (BHT) and acetyl bromide products were purchased from Sigma-Aldrich and used as received without further purification. (±)-3,6-dimethyl-1,4-dioxan-2,5-dione (DL-lactide) was also obtained from Sigma-Aldrich and recrystallised from ethyl acetate prior to use. Dimethylformamide (DMF) was purchased from Labkem. Dialysis membranes (MWCO 3.5 kDa, Spectra/Por®) and HPLC grade THF were bought Fisher Scientific. NMR solvents were purchased from Eurisotop.

The ^{1}H-NMR spectra were recorded on a Bruker 400 MHz spectrometer equipped with a CryoProbe Prodigy. All spectra were acquired in deuterated chloroform (CD_3Cl), using 32 scans and referenced internally to the solvent peak (δ = 7.26 ppm).

Gel-permeation chromatography (GPC) analyses of PEG-*b*-PLA diblock copolymers were performed in HPLC grade THF containing 250 ppm of BHT (1 ml/min) using an Agilent system equipped with a G7129A autosampler. Detection was carried out using a triple detector setup (1260 Agilent) comprising refractive index (RI), 2 angles Light Scattering (LS) operating at a wavelentgh of 658 nm and Viscosimeter. A PLgel 5 µm Mixed D 300-7.5 mm column with a molecular range 200-400000 Da was used for separation, which was used in a column heater at 30 ºC. The system was calibrated with a PS standard (Agilent).

Dynamic light scattering (DLS) measurements of PEG-*b*-PLA micelles were performed using the Zetasizer Ultra from Malvern Panalytical. Data were collected at a scattering angle of 173º using an avalanche photodidode detector and processed with ZSXPLORER software. Prior to measurements, samples were dialysed against Milli-Q water using a pre-wetted 3.5 kDa regenerated cellulose dialysis membrane.

Asymmetric flow field-flow fractionation analysis of PEG-*b*-PLA micelles were performed in aqueous solution containing 0.02% NaN_3 using a AF2000 MultiFlow system (Postnova Analytics). Detection was achieved using RI concentration detector (PN3150) and 18-angle LS detector (PN3621) operating at 532 nm for nanoparticle mass characterisation. Injections were done in triplicates. The specific refractive index increment (dn/dc), was determined for every sample being 0.041, 0.044, 0.044 and 0.05 for PEG_{46}-*b*-PLA_{16}, PEG_{114}-*b*-PLA_{40}, PEG_{228}-*b*-PLA_{80}, PEG_{456}-*b*-PLA_{120}, respectively.

Cryo-TEM imaging was performed on a Tecnai F20 Cryo-TEM instrument (FEI, The Netherlands), equipped with a Gatan Cryo-holder (Gatan, USA), at the Cryomicroscopy unit of the Scientific and Technological Centers from Universitat de Barcelona. For sample perparation, 1.5 µL of the dispersion was deposited onto a glow-discharged Lacey Carbon 300 mesh copper grid (Ted Pella, USA). Grids were maintained at 100% relative humidity and ambient temperature within a Vitrobot Mark III system (FEI, The Netherlands) during preparation. Excess of liquid was automatically blotted using filter paper, followed by rapid vitrification by plunge-freezing into liquid ethane. The vitrified samples were examined in cryogenic conditions at 200 kV and using low-dose imaging mode. The images were recorded with a 5096 x 4096 pixels CCD Eagle camera (FEI, The Netherlands).

Theoretical background and derivations of micelle radius (R_m)

As expressed in equation (1), the micelle radius (R_m) can be decomposed into the sum of the corona thickness (h_p), which depends on PEG degree of polymerisation (*N)* and the core radius (R_c), which depends on PLA degree of polymerisation (*M*). In principle, this leads to a two-variable description of R_m. However, since the PEG–PLA copolymers were systematically synthesised with a constant hydrophilic–hydrophobic ratio, Rm can be reformulated as a function of a single variable. Without loss of generality, we choose the degree of polymerisation of the PLA block as this independent parameter. Thus, given that the molar fraction of PLA (f_M) is fixed at 1/4, the following substitution can be made

$$f_M = \frac{M}{N+M} \implies 3M = N \qquad \text{(S1)}$$

Enabling to rewrite equation (2) as:

$$h_p(M) = R_c \left[\left(1 + \frac{(\gamma+2)M}{R_c} \left(\frac{va^2}{3\alpha_0} \right)^{\frac{1}{3}} \right)^{\frac{3}{\gamma+2}} - 1 \right] \qquad \text{(S2)}$$

As described in Tian *et al.*, the packing parameter can be taken as γ = 3 for high-curvature spherical micelles with tightly packed chains. Moreover, the monomer excluded volume can be approximated as $v \sim a^3$ (1). Therefore, equation (S1) can be simplified as:

$$h_p(M) = R_c \left[\left(1 + \frac{5M}{R_c} \left(\frac{a^5}{3\alpha_0} \right)^{\frac{1}{3}} \right)^{\frac{3}{5}} - 1 \right] \qquad \text{(S3)}$$

Hereafter, the derivation of the micelle radius (R_m) for each polymer chain model follows a consistent multi-step approach. The process begins with the expression of the aggregation number (Λ) as a function of the contour length of the hydrophobic PLA block. This expression is then used to derive the grafting density and the grafting surface. The grafting surface is subsequently inserted into the expression for the corona thickness (h_p), which accounts for the stretching of the hydrophilic PEG block. Finally, the value of h_p is incorporated into the equation for the total micelle radius (equation 1).

This general route, linking molecular parameter to the observable micelle radius, provides a modular framework that can be applied across all conformational regimes considered in this study. The following sections present the detailed derivations for each case.

2.1. Self-avoiding walk model

$$R_c = \langle r^2 \rangle^{\frac{1}{2}} = bM^{\nu} \quad \text{(S4)}$$

Extracted from experimental data, the aggregation number plays a central role in connecting the spatial configuration of the hydrophobic blocks to the overall micelle structure. The aggregation number can be defined in two complementary ways: first, as the ratio of the micelle number-average molecular weight M_n to that of a single polymer chain m_n (equation S5); and second, as the ratio of the total micelle core volume V_c to the volume occupied by a single polymer chain in the core,u_{PLA} (equation S6) :

$$\Lambda = \frac{M_n^{micelle}}{m_n^{unimer}} \quad \text{(S5);}$$

$$\Lambda = \frac{V_c}{u_{PLA}} \quad \text{(S6);}$$

For equation (S6), the numerator V_c and denominator u_{PLA} can be expressed as:

$$V_c = \frac{4}{3}\pi R_c^3 \quad \text{(S7);}$$

$$u_{PLA} = \frac{Mm_0}{\rho N_A} \quad \text{(S8),}$$

where in equation (S8), m_0 refers to the molecular weight of the lactic acid repeating unit, ρ to the PLA mass density, and N_A is Avogadro's constant.

By expressing R_c using its definition from equation (S4) and replacing both equations (S7) and (S8) into (S6), we rewrite the aggregation number as a function of the degree of polymerisation of the PLA block and its conformational state within the micelle core:

$$\Lambda(M) = \Phi M^{(3\nu - 1)} \quad \text{(S10),}$$

where the dimensionless pre-factor is defined as $\Phi = 4\pi\rho N_A b^3 / 3m_0$.

The derived equation (S10) is rather useful because it enables the extraction of conformational information about the PLA chain block from the experimental MALS data. Specifically, it allows us to estimate the effective Flory exponent. As shown in Figure S1, the experimentally derived exponents exhibit a clear trend across the series. For micelles formed by long PLA blocks (*i.e.,* PEG_{228}-*b*-PLA_{80} and PEG_{456}-*b*-PLA_{820}), the Flory exponent is close to 0.588, indicating that PLA chains adopt coiled conformations resembling those attained in a good solvent (2, 3). In contrast, for micelles with short PLA blocks (*i.e.,* PEG_{46}-*b*-PLA_{16} and PEG_{114}-*b*-PLA_{40}), the Flory exponents are significantly higher (0.73 and 0.68, respectively), suggesting more extended, radially oriented conformation within the core. This deviation, however, can be explained by considering the effects of spherical confinement and packing frustration of PLA chains within the micelle core (4, 5). Under such conditions, the conformational statistics of the hydrophobic block can be altered, and the SAW model no longer provides an adequate description. It is noteworthy that, despite the differences in size, the micelles across the series are composed of approximately the same number

of polymer chains. This has several implications. First, the interfacial area per chain at the core-corona boundary is not conserved across the series. As the micelle core grows with increasing PLA block length, both total surface area and area per chain increase as the aggregation number Λ is maintained. This enlarged interfacial area reduces steric crowding at the interface and allows the PEG corona chains to adopt more relaxed (6), less stretched conformations.

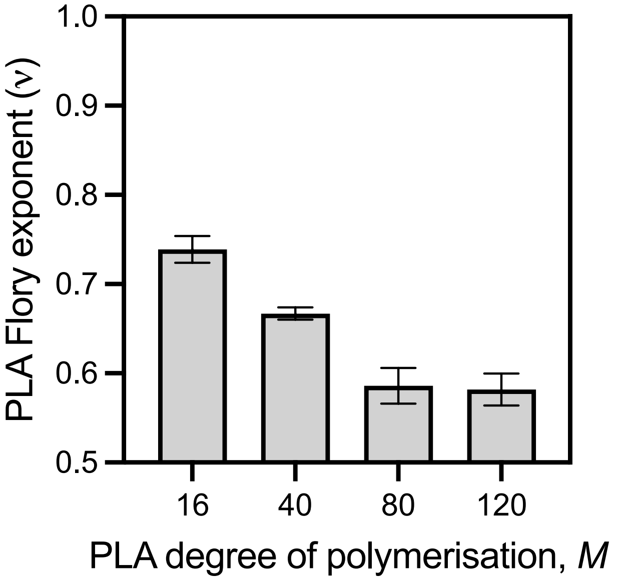

Figure S1. Flory exponent (ν) derived from the aggregation numbers of PEGn-*b*-PLAm calculated experimentally highlighting the variation in chain conformation across the micelle series.

2.2. Semi-flexible regime

$$R_c^2 = \langle r^2 \rangle = 2L_pL - 2L_p^2(1 - e^{-L/L_p}) \text{ (S4)}$$

From equation (S5) and (S6), we can rewrite equation (S10) as:

$$\Lambda(M) = \frac{V_c}{u_{PLA}} = \frac{4\pi\rho N_A}{3m_0}\frac{1}{M}R_c^3 = J\frac{1}{M}R_c^3 \text{ (S11)},$$

where $J = 4\pi\rho N_A/3m_0$ and it has dimensions of nm^{-3}.

Equation (S11) can hence be rewritten as a function of persistence length as ($M = L/b$):

$$\Lambda(L) = J\frac{b}{L}R_c^3 \text{ (S12)}$$

substituting R_c for the definition of the end-to-end chain length for the semi-flexible regime of the WLC model, leads to:

$$\Lambda(L) = J\frac{b}{L}\left[2L_pL - 2L_p^2(1 - e^{-L/L_p})\right]^{3/2} \text{ (eq. 5)},$$

Since $R_\mathrm{c}^2 = \langle r^2 \rangle = 2L_pL - 2L_p^2(1 - e^{-L/L_p})$.

The grating density from equation can also be rewritten by substituting R_c for the definition of the end-to-end chain length for the semi-flexible regime of the WLC model:

$$\sigma(L) = \frac{\Lambda}{4\pi\left[2L_pL - 2L_p^2(1 - e^{-L/L_p})\right]} \text{ (S13)}.$$

Substituting equation eq. (5) into (S13) leads to:

$$\sigma(L) = \frac{Jb}{4\pi L}\frac{\left[2L_pL - 2L_p^2(1 - e^{-L/L_p})\right]^{3/2}}{\left[2L_pL - 2L_p^2(1 - e^{-L/L_p})\right]} = \frac{Jb}{4\pi L}\left[2L_pL - 2L_p^2(1 - e^{-L/L_p})\right]^{3/2} \cdot \left[2L_pL - 2L_p^2(1 - e^{-L/L_p})\right]^{-1}$$

$$= \frac{Jb}{4\pi L}\left[2L_pL - 2L_p^2(1 - e^{-L/L_p})\right]^{1/2} = \frac{Jb}{4\pi L}R_\mathrm{c} \text{ (S14)}$$

The substitution of equation (S14), through the relationship of $\sigma = \alpha_0^{-1}$ into equation (S3), expressed as function of the contour length *L,* leads to:

$$h_\mathrm{p}(L) = R_\mathrm{c}\left[\left(1 + \frac{5}{b}\frac{L}{R_\mathrm{c}}\left(\frac{a^5}{3}\frac{Jb}{4\pi L}R_\mathrm{c}\right)^{\frac{1}{3}}\right)^{\frac{3}{5}} - 1\right] \text{ (S15)},$$

which can be simplified and reorganised as:

$$h_\mathrm{p}(L) = R_\mathrm{c}\left[\left(1 + 5\left(\frac{a^5J}{12\pi}\right)^{1/3}\left(b^{1/3}\cdot b^{-1}\right)\left(L^{-1/3}\cdot L\right)\left(R_\mathrm{c}^{1/3}\cdot R_\mathrm{c}^{-1}\right)\right)^{3/5} - 1\right] = R_\mathrm{c}\left[\left(1 + \Omega\cdot L^{2/3}\cdot R_\mathrm{c}^{-2/3}\right)^{3/5} - 1\right] \text{ (S16)}$$

Where $\Omega = 5\cdot b^{-2/3}\left(\frac{a^5J}{12\pi}\right)^{1/3}$.

Substituting equation (S4) into (S16) leads to:

$$h_{\mathrm{p}}(L) = \left[2L_pL - 2L_p^2(1 - e^{-L/L_p})\right]^{1/2}\left[\left(1 + \Omega\cdot L^{2/3}\cdot\left[2L_pL - 2L_p^2(1 - e^{-L/L_p})\right]^{-1/3}\right)^{3/5} - 1\right] \quad \text{(S17)}$$

Finally, substituting equation (S17) into equation (1) and simplifying, leads to the final expression of R_{m} for the semi-flexible regime of the WLC model:

$$R_{\mathrm{m}}(L) = F^{1/2}\left(1 + \Omega\cdot L^{2/3}\cdot F^{-1/3}\right)^{\frac{3}{5}} \quad \text{(S18)}$$

where $F = 2L_pL - 2L_p^2(1 - e^{-L/L_p})$

2.4. Flexible regime

The original expression (S4) assessed in the $L \gg L_p$ regime, *i.e., $L/L_{\mathrm{p}} \gg 1$*, can be simplified as:

$$R_{\mathrm{c}}^2 = \langle r^2\rangle \approx 2L_{\mathrm{p}}L \quad \text{(S4a)},$$

Thus, from equation (S12) and equation (S4a), we can derive an expression for the aggregation number as a function of the contour length for the WLC model in the flexible regime:

$$\Lambda(L) = J\frac{b}{L}\left(2L_{\mathrm{p}}L\right)^{3/2} = Jb(2L_{\mathrm{p}})^{3/2}L^{1/2} \quad \text{(eq. 6)}$$

As from the final expression for equation (S14), we can derive the expression of the grafting density as a function of the contour length for the flexible regime in the WLC model as:

$$\sigma(L) = \frac{Jb}{4\pi L}R_{\mathrm{c}} = \frac{Jb}{4\pi L}\left(2L_{\mathrm{p}}L\right)^{1/2} \quad \text{(S19)}$$

which can be simplified and reorganised as:

$$\sigma(L) = \frac{Jb}{4\pi L}\left(2L_{\mathrm{p}}L\right)^{1/2} = \frac{Jb\sqrt{2}}{4\pi}L_{\mathrm{p}}^{1/2}L^{1/2}L^{-1} = Cb\left(\frac{L_{\mathrm{p}}}{L}\right)^{1/2} \quad \text{(S20)},$$

Where $C = J\sqrt{2}/4\pi$.

The substitution of equation (S20), through $\sigma = \alpha_0^{-1}$, into equation (S3), expressed as function of the contour length *L,* leads to:

$$h_{\mathrm{p}}(L) = R_{\mathrm{c}}\left[\left(\left(1+\frac{5}{b}\frac{L}{R_{\mathrm{c}}}\left(\frac{a^5}{3}Cb\left(\frac{L_p}{L}\right)^{1/2}\right)^{1/3}\right)\right)^{3/5} - 1\right] \quad \text{(S21)}$$

Which can be rearranged as:

$$h_{\mathrm{p}}(L) = R_{\mathrm{c}}\left[\left(1+\frac{5}{b}\left(\frac{a^5}{3}Cb\right)^{1/3}\cdot\frac{L}{R_{\mathrm{c}}}\left(\frac{L_p}{L}\right)^{1/6}\right)^{3/5} - 1\right] \quad \text{(S22)}$$

Substituting equation (S4a) into (S22) yields:

$$h_{\mathrm{p}}(L) = \sqrt{2L_{\mathrm{p}}L}\left[\left(1+\frac{5}{b}\left(\frac{a^5}{3}Cb\right)^{1/3}\cdot\frac{L}{\sqrt{2L_{\mathrm{p}}L}}\left(\frac{L_p}{L}\right)^{1/6}\right)^{3/5} - 1\right] \quad \text{(S23)}$$

Which can be rearranged as:

$$h_{\mathrm{p}}(L) = \sqrt{2L_{\mathrm{p}}L}\left[\left(1+\Gamma\cdot\left(L\cdot L^{1/2}\cdot L^{-1/6}\right)\left(L_{\mathrm{p}}^{1/6}\cdot L_{\mathrm{p}}^{-1/2}\right)\right)^{3/5} - 1\right] = \sqrt{2L_{\mathrm{p}}L}\left[\left(1+\Gamma\cdot\frac{L^{1/3}}{L_{\mathrm{p}}^{2/3}}\right)^{3/5} - 1\right] \quad \text{(S24)}$$

Where $\Gamma = \frac{5}{\sqrt{2}b}\left(\frac{a^5}{3}Cb\right)^{1/3}$.

Finally, substituting equation (S24) into equation (1) and simplifying, leads to the final expression of R_{m} for the flexible regime of the WLC model:

$$R_{\mathrm{m}}(L) = \sqrt{2L_pL}\left(1+\Gamma\frac{L^{1/3}}{L_p^{2/3}}\right)^{\frac{3}{5}} \quad \text{(S25)}$$

2.5. Rigid-rod regime

The original expression (S4) assessed in the $L \ll L_p$ regime, *i.e.,* $L/L_{\mathrm{p}} \ll 1$, can be simplified as:

$$R_{\mathrm{c}}^2 = \langle r^2\rangle \approx L^2 - \frac{L^3}{3L_{\mathrm{p}}} \quad \text{(S4b),}$$

by using the Taylor series around zero:

$$e^{-L/L_\mathrm{p}} \approx 1 - \frac{L}{L_\mathrm{p}} + \frac{1}{2}\left(\frac{L}{L_\mathrm{p}}\right)^2 - \frac{1}{6}\left(\frac{L}{L_\mathrm{p}}\right)^3 + \ldots + \frac{1}{n!}\left(\frac{L}{L_\mathrm{p}}\right)^n$$

Thus, from equation (S12) and equation (S4b), we can derive an expression for the aggregation number as a function of the contour length for the WLC model in the rigid-rod regime:

$$\Lambda(L) = J\frac{b}{L}\left(L^2 - \frac{L^3}{3L_P}\right)^{3/2} \quad \text{(S26)}$$

where J has already been defined for the semi-flexible regime.

As from the final expression for equation (S14), we can derive the expression of the grafting density as a function of the contour length for the rigid regime in the WLC model as:

$$\sigma(L) = \frac{Jb}{4\pi L}R_\mathrm{c} = \frac{Jb}{4\pi L}\left(L^2 - \frac{L^3}{3L_p}\right)^{1/2} \quad \text{(S27).}$$

The substitution of equation (S27), through the relationship $\sigma = \alpha_0^{-1}$, into equation (S3), expressed as function of the contour length L, leads to:

$$h_\mathrm{p}(L) = \left(L^2 - \frac{L^3}{3L_P}\right)^{1/2}\left[\left(1 + \Omega \cdot L^{2/3} \cdot \left(L^2 - \frac{L^3}{3L_P}\right)^{-1/3}\right)^{3/5} - 1\right] \quad \text{(S28),}$$

Where Ω has already been defined for the semi-flexible regime.

Finally, substituting equation (S28) into equation (1) and simplifying, leads to the final expression of R_m for the rigid-rod regime of the WLC model:

$$R_\mathrm{m}(L) = Q^{1/2}\left(1 + \Omega \cdot L^{2/3} \cdot Q^{-1/3}\right)^{3/5} \quad \text{(S29)}$$

where $Q = L^2 - \dfrac{L^3}{3L_p}$.

Molecular dynamics simulations of PEG_{46}-PLA_{16} Micelle

We built a single linear PEG_{46}-PLA_{16} topology using the MARTINI force field, using a single bead to model each PEG or PLA monomer as previously used in López-Rios de Castro et al. (7).The linear monomer was initially simulated in an empty simulation box of volume 100x100x100 nm^3 to reach an equilibrium conformation. All the following molecular dynamics (MD) simulations were conducted using Gromacs 2021.2 (8). We first run an energy minimization using the steepest-descent algorithm with a tolerance of 10 $kJ \cdot mol^{-1} \cdot nm^{-2}$. The van der Waals force has a cut-off at 1.1 nm. The long-range electrostatic interactions were computed using the reaction field algorithm (9) with the cutoff distance set to 11 Å. We set the relative dielectric constant to 15. The algorithm LINCS manages the constraints. We then performed an equilibration simulation using the NVT ensemble for 6 ns using a timestep of 20 fs. The temperature was controlled using the velocity-rescale thermostat with 1 ps coupling constant and a target temperature of 298.15K. The coiled PEG_{46}-PLA_{16} was replicated in Chimera (10) to create a cubic box with each side being 24 nm in length containing 46 polymers. The box was solvated with MARTINI 2 polarizable water (11), which resulted in a system containing around 330 000 particles. After a first minimization, using the same parameters as for the single polymer in vacuum, but changed the relative dielectric constant was changed to 2.5, we conducted an NVT equilibration run of 1 ns generating particle velocities from the Maxwell distribution at 303.15K and using the timestep of 20 fs. We again used the velocity rescale thermostat with coupling constant of 1 ps. Then, we equilibrated the volume in a successive simulation using the NPT ensemble for 10 ns, using the C-rescale isotropic barostat with a coupling constant of 5 ps, a compressibility of $4.5 x 10^{-5}$ bar^{-1} and a target pressure of 1 bar. We then let the polymers aggregate during a 1.1 µs long simulation employing the NVT ensemble. This resulted in two aggregates each containing 23 PEG_{46}-PLA_{16} polymers. In order to overcome the timescales that would be required to observe these two aggregates to self-assemble into one large micelle, we applied a pulling force between the two PLA cores using a constant attractive force of 500 kJ mol^{-1} nm^{-2}. After 10 ns employing the NPT ensemble, the two smaller aggregates merged into a single PEG_{46}-PLA_{16} micelle containing all 46 molecules. After forming this initial structure for the PEG_{46}-PLA_{16} micelle with the experimentally observed aggregation number, we finally equilibrated the micelle components within a 0.15 M NaCl solution using a 50 ns molecular dynamics simulation employing the NVT ensemble. The analysis of the production trajectory was conducted using in-house Python script which utilised the MDAnalysis python package (12).

Synthesis of PEG-*b*-PLA diblock copolymers

In a flame-dried Schlenk under argon atmosphere, mPEG-OH (1 equivalent) and DL-lactide (8 equivalent for PEG_{46}-*b*-PLA_{16}, 20 equivalent PEG_{114}-*b*-PLA_{40}, 40 equivalent PEG_{228}-*b*-PLA_{80} and 80 equivalent for PEG_{456}-*b*-PLA_{120}) were dissolved in the minimum amount of anhydrous THF and stirred for an hour. Next, THF was removed under reduced pressure to facilitate the azeotropic drying of mPEG-OH via THF/water azeotrope. Once the solvent was completely evaporated and a dry powder was obtained, the mixture was further dried under vacuum for an additional hour. Subsequently, anhydrous DCM was added to the flask to reach a final DL-lactide concentration of 0.1 g/ml. Polymerisation was initiated by the addition of 1%mol DBU relative to DL-lactide. The reaction mixture was stirred at room temperature for 3 hours. Polymerisation was quenched by the addition of 5 equivalents of acetyl bromide, relative to the hydroxyl end-groups, to promote end-group esterification and 5 equivalents of DBU as base. The reaction was allowed to proceed

overnight under continuous stirring. The resulting polymer was purified by three cycles of precipitation into liquid-nitrogen cooled methanol, followed by centrifugation. The final product was collected and dried under a vacuum oven. It was then redissolved in deionised water and subjected to freeze-drying, yielding a white, cotton-like powder.

Micellization of PEG-*b*-PLA diblock copolymers

Solutions of PEG-*b*-PLA (10 mg/ml) were prepared in DMF. A volume of 0.6 µL was injected into 3.4 mL of vigorously stirred Milli-Q water at a flow rate of 100 µL/min using a NE-4002X syringe pump. The resulting dispersion was immediately transferred into a dialysis membrane (MWCO 3.5 kDa) and dialysed against Milli-Q water to remove residual solvent. The external water was replaced three times to ensure complete solvent exchange.

Table S1 — Summary table of constants and PLA monomer size estimation

Parameter	Symbol	Value
PEG monomer length	a	0.30 nm
PLA monomer length	b	0.37 nm
PLA molecular weight	m_0	72 g/mol
PLA monomer density	ρ	1.20 g/cm^3

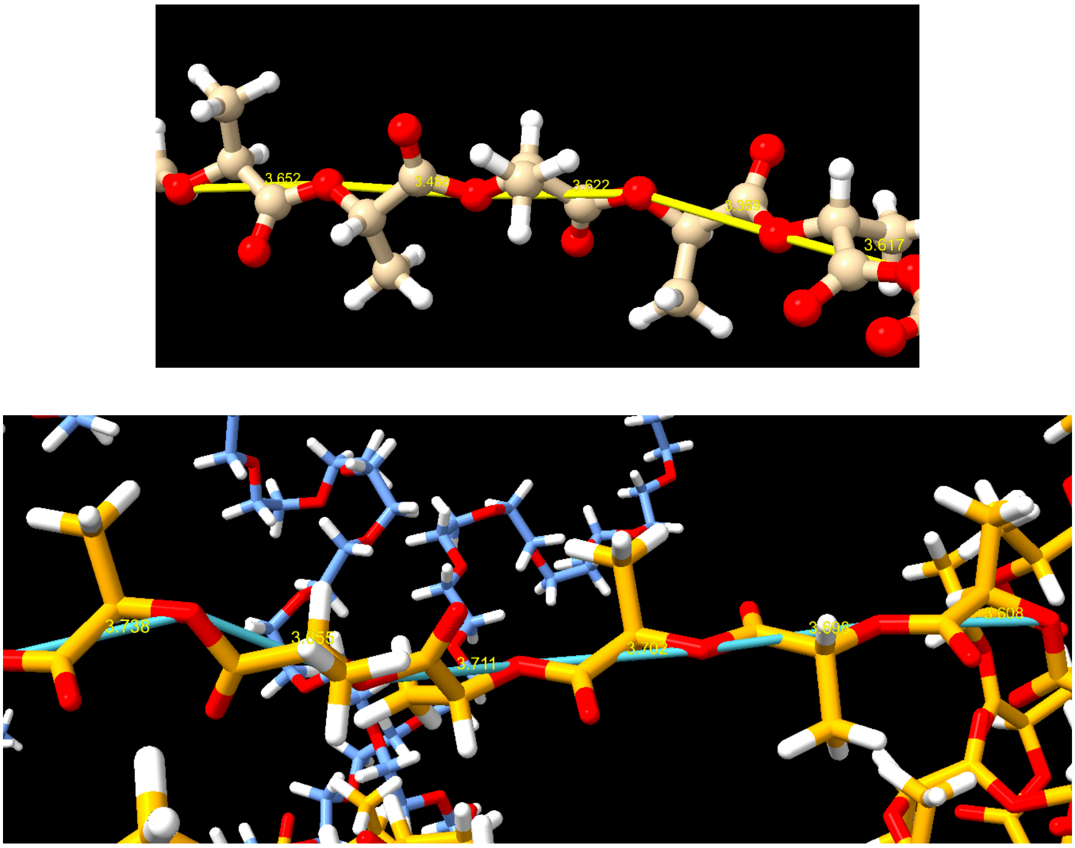

Figure S2. Snapshots illustrating the determination of PLA monomer dimensions, in Å, using Avogadro molecular modelling software.

^{1}H-NMR spectrum of PEG_{46}-PLA_{16}

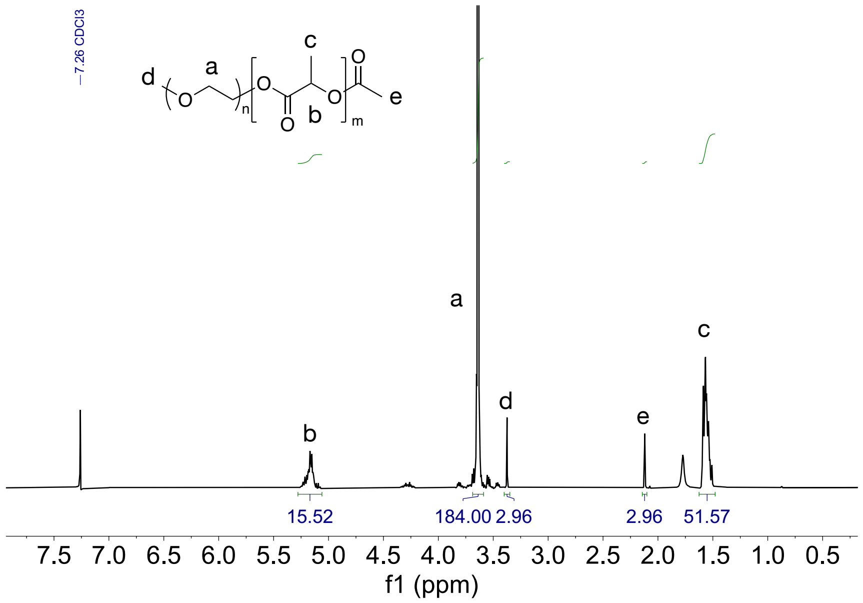

Figure S3 ^{1}H-NMR spectrum of PEG_{46}-*b*-PLA_{16} recorded in $CDCl_3$ on a Bruker 400 MHz spectrometer. Chemical shifts were internally referenced to the residual solvent signal (δ = 7.26 ppm). Signal integrals were normalised based on the known number of protons in the mPEG block.

^{1}H-NMR spectrum of PEG_{114}-PLA_{40}

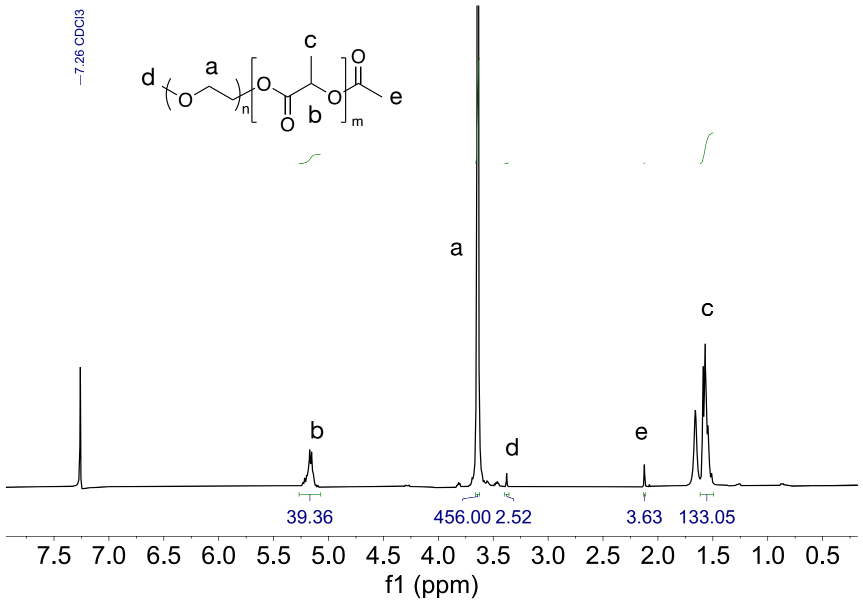

Figure S ^{1}H-NMR spectrum of PEG_{114}-*b*-PLA_{40} recorded in $CDCl_3$ on a Bruker 400 MHz spectrometer. Chemical shifts were internally referenced to the residual solvent signal (δ = 7.26 ppm). Signal integrals were normalised based on the known number of protons in the mPEG block.

^{1}H-NMR spectrum of PEG_{228}-PLA_{80}

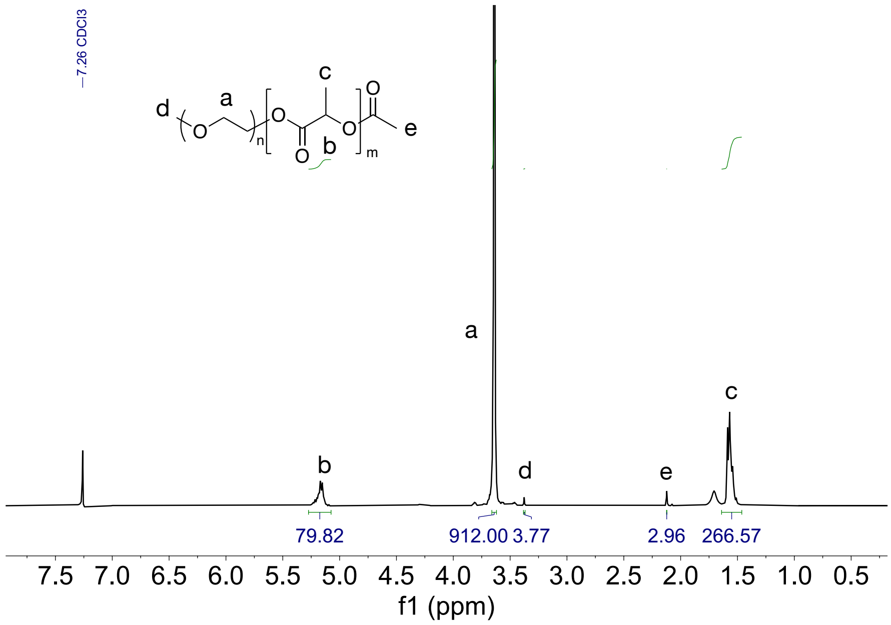

Figure S5 ^{1}H-NMR spectrum of PEG_{228}-*b*-PLA_{80} recorded in $CDCl_3$ on a Bruker 400 MHz spectrometer. Chemical shifts were internally referenced to the residual solvent signal (δ = 7.26 ppm). Signal integrals were normalised based on the known number of protons in the mPEG block.

^{1}H-NMR spectrum of PEG_{456}-PLA_{120}

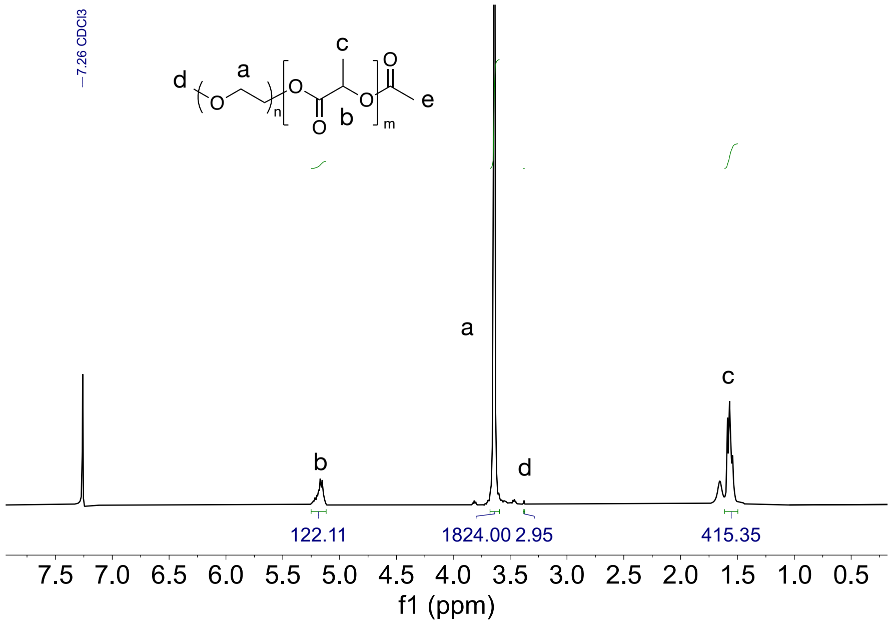

Figure S6 ^{1}H-NMR spectrum of PEG_{456}-*b*-PLA_{120} recorded in $CDCl_3$ on a Bruker 400 MHz spectrometer. Chemical shifts were internally referenced to the residual solvent signal (δ = 7.26 ppm). Signal integrals were normalised based on the known number of protons in the mPEG block.

Figure S7 — GPC traces of PEG-PLA polymer chains

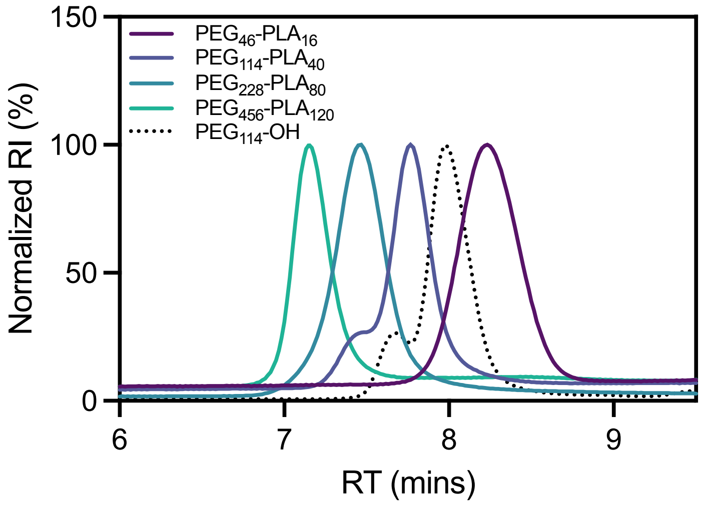

Figure S7. GPC traces of PEG–PLA diblock copolymers. The solid lines correspond to the PEG–PLA samples, while the dotted line represents the PEG_{114}-OH macroinitiator used for PEG_{114}–PLA_{40} synthesis. A clear shift toward lower elution volumes (higher molecular weights) confirms the successful chain extension of PEG with the PLA block.

GPC molecular weight distributions of PEG-PLA polymer chains

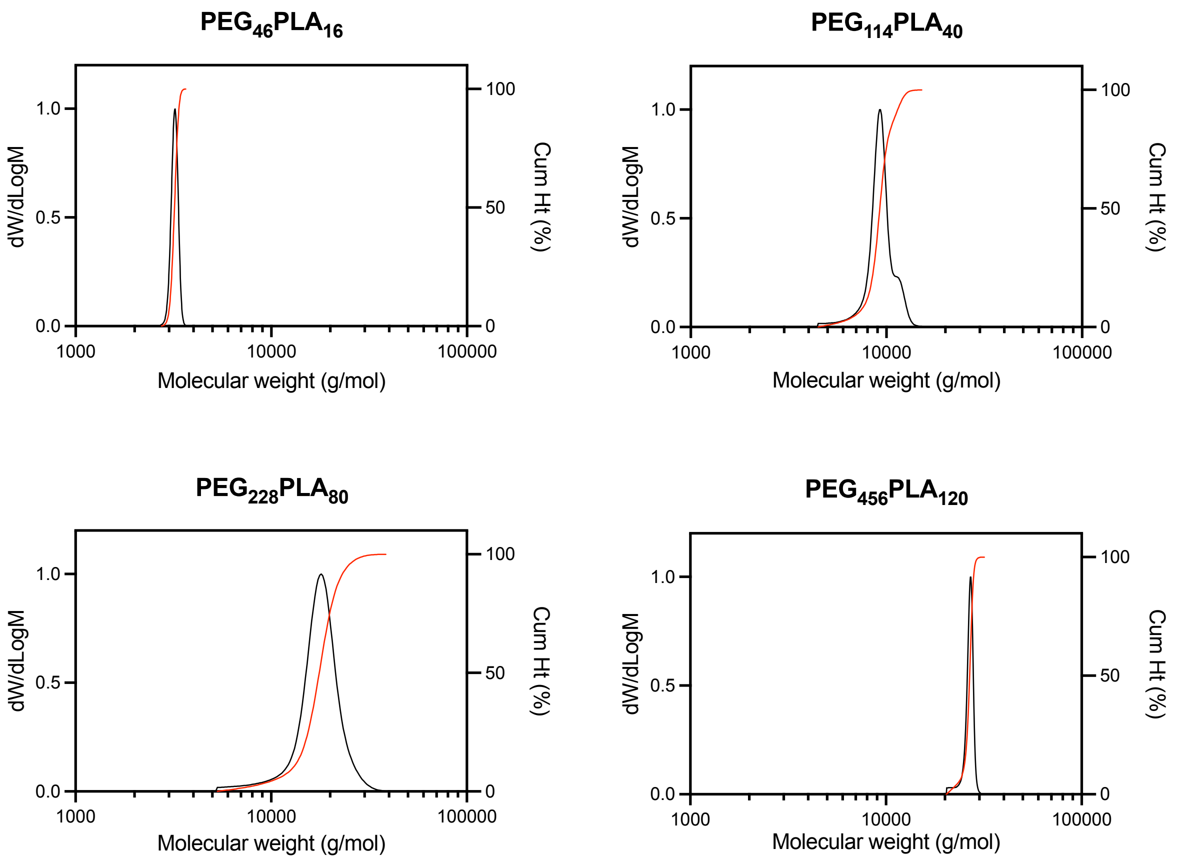

Figure S8. The black curve represents the differential weight distribution (*dw*/*d*Log*M*), while the red curve shows the cumulative weight fraction (cumulative height). Mw corresponds to the point where the cumulative curve reaches 50% of the total polymer mass, whereas M_n is defined as the molecular wight at which an equal number of chains lie on either side of the distribution. The steepness of the cumulative curve reflects the polydispersity of the sample, the steeper the transition, the closer M_n and M_w values are, indicating a narrower, more monodisperse molecular weight distribution.

PEG-PLA micelles CryoTEM size distributions

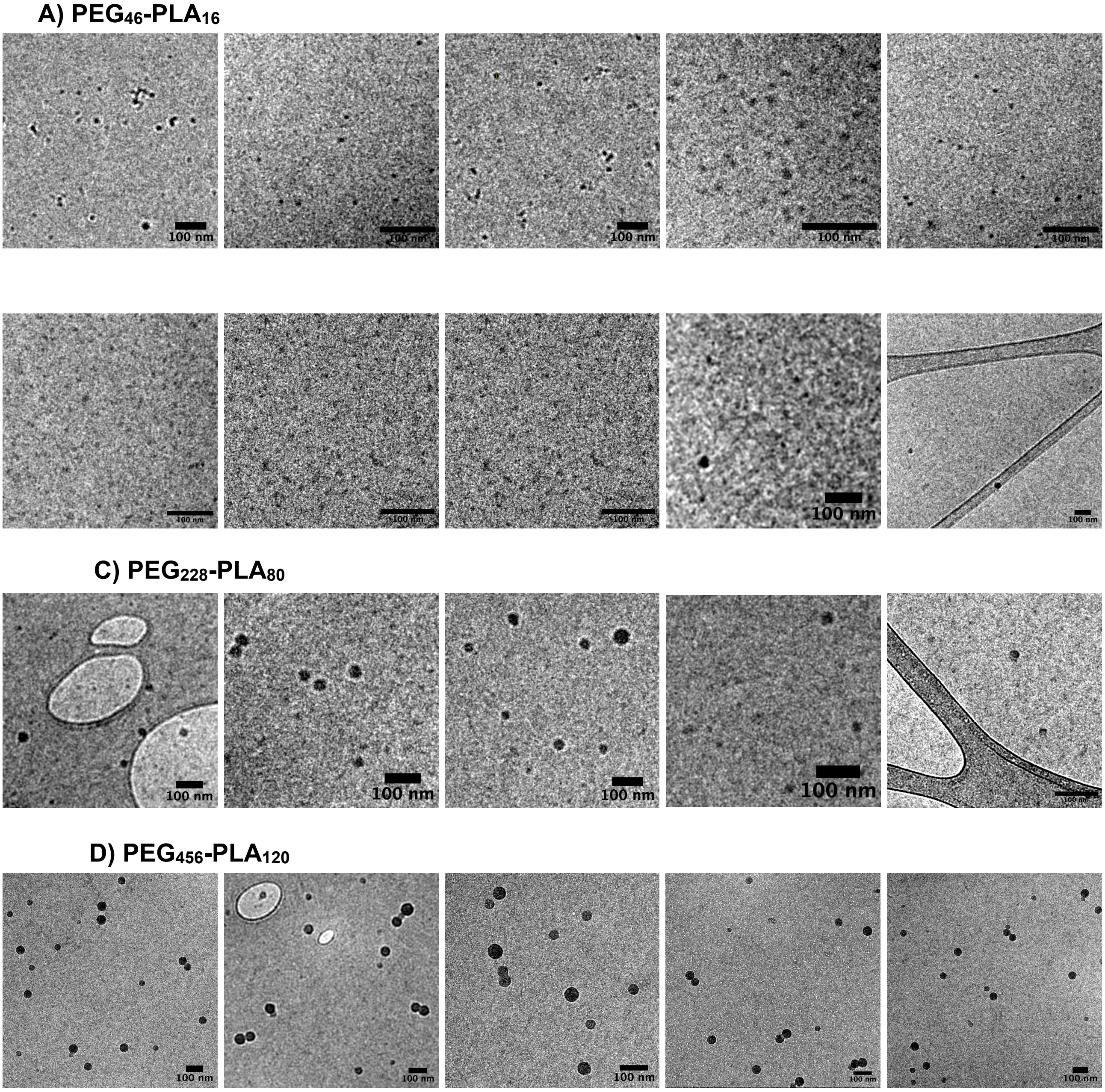

Figure S9. CryoTEM micrographs of A) $PEG_{46}PLA_{16}$, B)$PEG_{114}PLA_{40}$, C)$PEG_{228}PLA_{80}$ and C) $PEG_{456}PLA_{120}$ micelles at different magnifications, revealing their spherical morphology and uniform size. All scale bars are equal to 100 nm.

Correlation between DLS and CryoTEM micelle radii

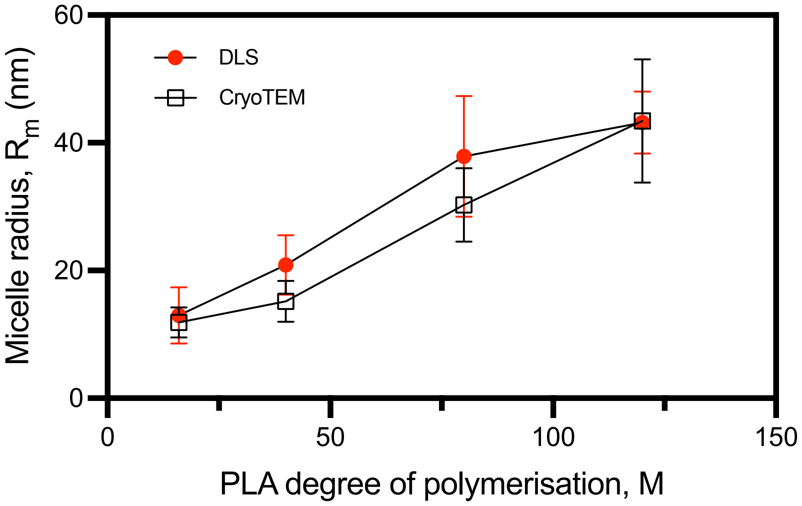

Figure S10. Comparison of micelle sizes determined by dynamic light scattering (DLS) and cryogenic transmission electron microscopy (CryoTEM)

Light scattering characterization and angular dependence of PEG-PLA micelles

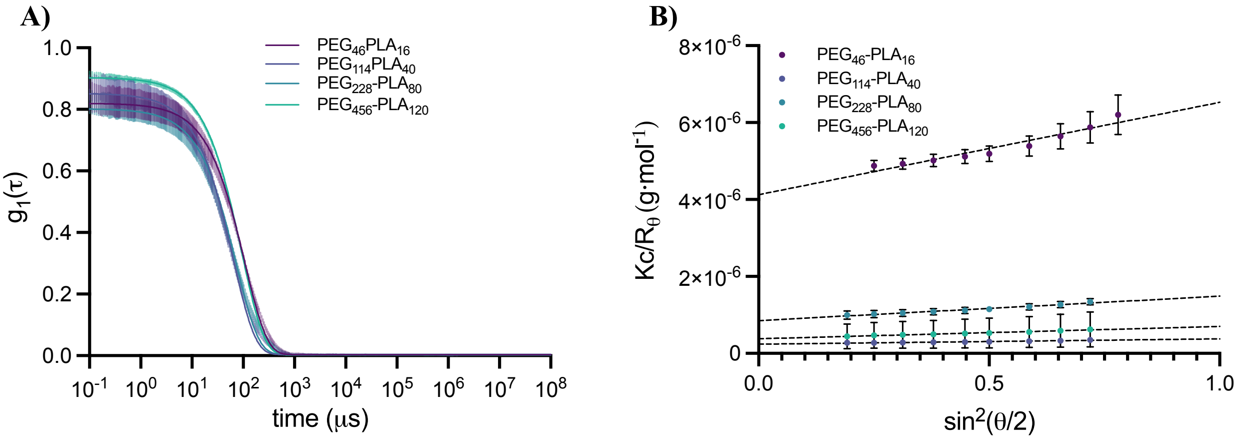

Figure S11. A) Correlation functions obtained from DLS measurements of the micelles. B) Corresponding Zimm plots showing the angular dependence of the scattering intensity.

AF4 molecular weight distributions of the micelle series

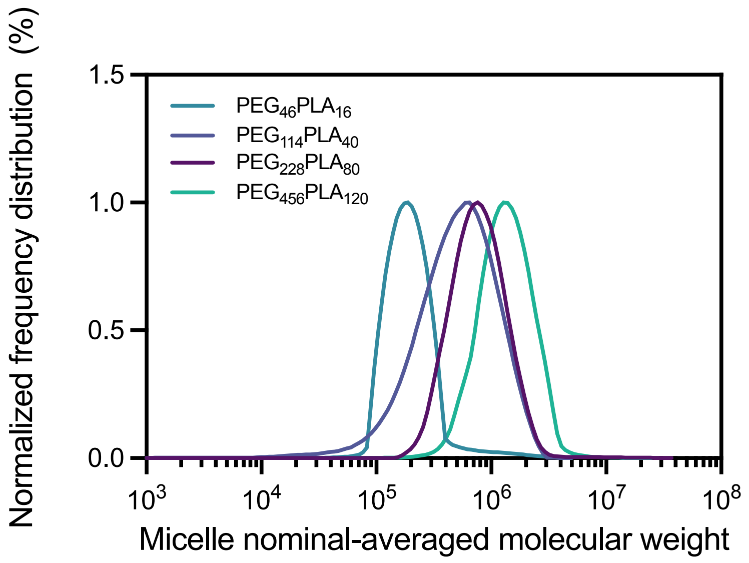

Figure S12. AF4-derived molecular weight distributions for the PEG-*b*-PLA micelles series, showing variation in molar mass and distribution profiles across different block copolymer formulations.

Rigid-rod and semi-flexible WLC fit

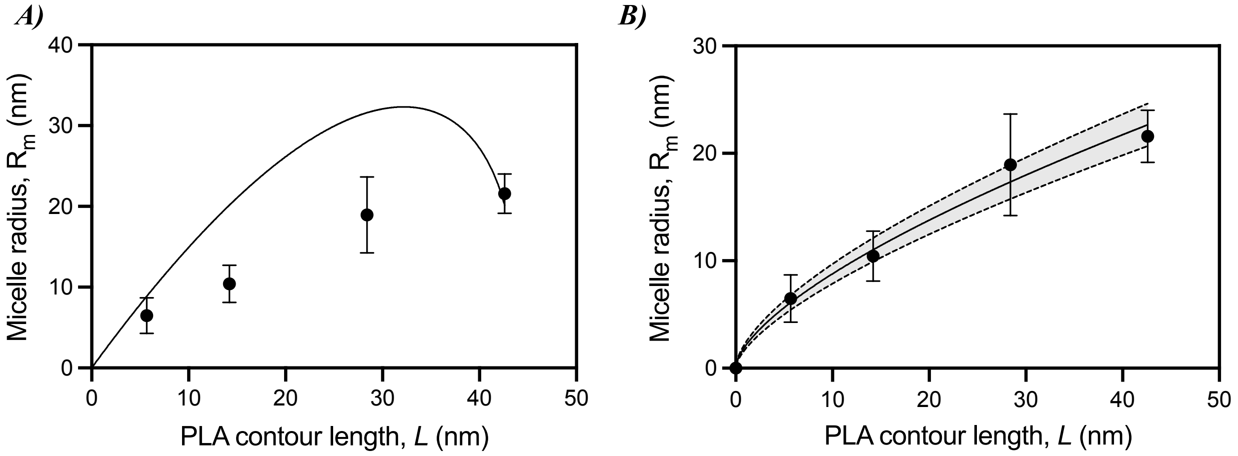

Figure S13. Nonlinear regression analysis of micelle series using the worm-like chain (WLC) model under two distinct conformational regimes (A) rigid-rod and (B) semi-flexible. While the semi-flexible regime provides a visually reasonable fit of the experimental data, the overall quality of the regression is inferior to that of the flexible regime (see main text).

Experimental determination of L_p for each micelle in the semi-flexible regime

The persistence length L_p of each PLA block in the micelle series was determined by numerically solving equation (5) using Brent's root-finding method, within a range comprehended between 10^{-3} and 10^{3} nm, both included

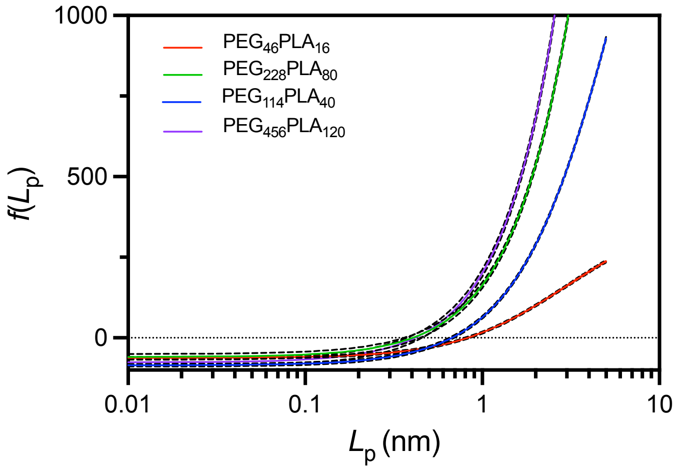

Figure S14. Determination of the individual persistence lengths L_p for each micelle using the WLC-derived expression for the aggregation number in the semi-flexible regime. The roots of the curves correspond to the calculated L_p values

Bibliography

1. Tian X, Angioletti-Uberti S, Battaglia G. On the design of precision nanomedicines. Science Advances. 2020;6(4):eaat0919.
2. Almdal K, Rosedale JH, Bates FS, Wignall GD, Fredrickson GH. Gaussian- to stretched-coil transition in block copolymer melts. Physical Review Letters. 1990;65(9):1112-5.
3. Zhulina EB, Adam M, LaRue I, Sheiko SS, Rubinstein M. Diblock Copolymer Micelles in a Dilute Solution. Macromolecules. 2005;38(12):5330-51.
4. Cacciuto A, Luijten E. Self-Avoiding Flexible Polymers under Spherical Confinement. Nano Letters. 2006;6(5):901-5.
5. Gao J, Tang P, Yang Y, Chen JZY. Free energy of a long semiflexible polymer confined in a spherical cavity. Soft Matter. 2014;10(26):4674-85.
6. Battaglia G, Ryan AJ. Bilayers and Interdigitation in Block Copolymer Vesicles. Journal of the American Chemical Society. 2005;127(24):8757-64.
7. Lopez-Rios de Castro R, Ziolek RM, Ulmschneider MB, Lorenz CD. Therapeutic Peptides Are Preferentially Solubilized in Specific Microenvironments within PEG-PLGA Polymer Nanoparticles. Nano Lett. 2024;24(6):2011-7.
8. Abraham MJ, Murtola T, Schulz R, Páll S, Smith JC, Hess B, et al. GROMACS: High performance molecular simulations through multi-level parallelism from laptops to supercomputers. SoftwareX. 2015;1-2:19-25.
9. Hess B, Kutzner C, van der Spoel D, Lindahl E. GROMACS 4: Algorithms for Highly Efficient, Load-Balanced, and Scalable Molecular Simulation. Journal of Chemical Theory and Computation. 2008;4(3):435-47.
10. Pettersen EF, Goddard TD, Huang CC, Couch GS, Greenblatt DM, Meng EC, et al. UCSF Chimera—A visualization system for exploratory research and analysis. Journal of Computational Chemistry. 2004;25(13):1605-12.
11. Yesylevskyy SO, Schäfer LV, Sengupta D, Marrink SJ. Polarizable Water Model for the Coarse-Grained MARTINI Force Field. PLOS Computational Biology. 2010;6(6):e1000810.
12. Michaud-Agrawal N, Denning EJ, Woolf TB, Beckstein O. MDAnalysis: A toolkit for the analysis of molecular dynamics simulations. Journal of Computational Chemistry. 2011;32(10):2319-27.
